# Supplementary material for: Leading wellness in healthcare: A qualitative study of leadership practices for wellness in hospital settings
Source: PLoS One. 2023 Jul 19;18(7):e0285103. doi: 10.1371/journal.pone.0285103 (PMC10355419; doi:10.1371/journal.pone.0285103)
Supplement: Attachment — Submitted filename: response to reviewers_PONE-D-23-00842.docx [file pone.0285103.s002.docx]

Response to reviewers

PONE-D-23-00842 - Leading wellness in healthcare: A qualitative study of leadership practices for wellness in hospital settings.

Thank you for the opportunity to revise and improve our manuscript. Responses are below and in tracked changes in the manuscript.

| ***Editor recommendations*** | |
| --- | --- |
| **Comment** | **Response** |
| Please remove any funding-related text from the manuscript and let us know how you would like to update your Funding Statement. | The funding statement has been removed from the manuscript |
| Competing interests statement | The competing interests statement has been updated in the cover letter to include - "This does not alter our adherence to PLOS ONE policies on sharing data and materials.” |
| Data availability statement | The cover letter has included an updated data availability statement. |
| Describe in more detail how practice theory informed the approach | Further elaboration of practice theory has been added on p.5 line 121-123 and how it informed the data analysis on p.8 lines 188-189 |
| Include a table of participant characteristics | A table has been added on p. 7-8 |
| Include more of a description of the co-analysis sessions; how were these sessions run, what was their content, etc. | An explanation of the co-analysis process has been added to p.9 line 195-203 |
| Better situate the findings from this study within the broader literature - this is briefly done in the Discussion (p.21) but should be expanded | Additional material has been added to the discussion on pp.20 and 22 to incorporate both the leadership and change literature and leadership practices literature. |
| Include the COREQ checklist as a supplemental file | Completed and uploaded |
| **Reviewer 1** | |
| 1) What were the experiences of the leaders in the study? For example, did most leaders in the study have over 5 or 10 years experience? The study results may differ depending on the experience of leaders. | Information about the leader roles and experience has been added on p.7 (years in healthcare) and in a participant demographics table on p.7-8. |
| 2) Try not to use the same quote twice (see page 10). | The repeated quote has been deleted p.11 |
| 3) Did the study look at wellness and its impact on staff motivation, patient quality and safety through leadership? It would be insightful to have this discussion. | No. A stepped wedge trial of SEED is planned if it is expanded to other sites. The following has been added to the strengths and limitations section. The impact of the SEED program and related leadership practices on staff motivation and patient quality and safety has not been evaluated. Further research is required to investigate the objective results of the SEED program. |
| 4) Innovations were not clear in the study. Perhaps give a few examples of the innovations resulted from leadership in SEED. | More detail on the SEED program and some of the activities arising from it are provided on p.7 |
| 5) Overall, the techniques shown by the leaders and healthcare workers were insightful in the study. The next study could look at the leadership skills in SEED impacting on staff morale, patient care, teamwork, and the organisation effectiveness. | Agreed. Further research is required and is planned if SEED continues. |
| **Reviewer 2** | |
| Thank you for giving me the opportunity to read this well written paper. The topic is highly relevant also for an international audience. | Thank you |
| Unfold what the SEED program consisted of, how the different versions developed (and continued) | See more detailed description on p.7 |
| Describe and argue in more depth for your qualitative approach (why different methods and approaches), | More detail has been added about practice theory in the introduction and data analysis sections. Additional information about the co-analysis has been added in the data analysis section. |
| Describe how the groups were combined (leaders only or mixed and why) | Further information on the data collection has been added on p.8 Data collection was conducted by site. Focus groups and co-analysis sessions included both leaders and healthcare workers who had participated in SEED. Because SEED is inclusive of all staff in a team who participate equally, data collection was not organised by role. |
